# Dynamic Network Drivers of Seizure Generation, Propagation and Termination in Human Neocortical Epilepsy

**DOI:** 10.1371/journal.pcbi.1004608

**Published:** 2015-12-17

**Authors:** Ankit N. Khambhati, Kathryn A. Davis, Brian S. Oommen, Stephanie H. Chen, Timothy H. Lucas, Brian Litt, Danielle S. Bassett

**Affiliations:** 1 Department of Bioengineering, University of Pennsylvania, Philadelphia, Pennsylvania, United States of America; 2 Penn Center for Neuroengineering and Therapeutics, University of Pennsylvania, Philadelphia, Pennsylvania, United States of America; 3 Department of Neurology, Hospital of the University of Pennsylvania, Philadelphia, Pennsylvania, United States of America; 4 Department of Neurosurgery, Hospital of the University of Pennsylvania, Philadelphia, Pennsylvania, United States of America; 5 Department of Electrical and Systems Engineering, University of Pennsylvania, Philadelphia, Pennsylvania, United States of America; UNITED STATES

## Abstract

The epileptic network is characterized by pathologic, seizure-generating ‘foci’ embedded in a web of structural and functional connections. Clinically, seizure foci are considered optimal targets for surgery. However, poor surgical outcome suggests a complex relationship between foci and the surrounding network that drives seizure dynamics. We developed a novel technique to objectively track seizure states from dynamic functional networks constructed from intracranial recordings. Each dynamical state captures unique patterns of network connections that indicate synchronized and desynchronized hubs of neural populations. Our approach suggests that seizures are generated when synchronous relationships near foci work in tandem with rapidly changing desynchronous relationships from the surrounding epileptic network. As seizures progress, topographical and geometrical changes in network connectivity strengthen and tighten synchronous connectivity near foci—a mechanism that may aid seizure termination. Collectively, our observations implicate distributed cortical structures in seizure generation, propagation and termination, and may have practical significance in determining which circuits to modulate with implantable devices.

## Author Summary

Localization-related epilepsy is a debilitating condition where seizures begin in dysfunctional brain regions, and is often resistant to medication. The challenge for treating patients is mapping connections between cortical structures that vary with time and drive seizure dynamics. While it is well known that whole-brain functional architecture reconfigures during tasks, we hypothesize that epileptic networks reconfigure at the meso-scale leading to seizure generation, propagation, and termination. We develop new methods to track dynamic network reconfiguration amongst connections of different strength as seizures evolve. Our results indicate that seizure generation is primarily driven by rapidly reorganizing weak connections that drive stronger connections to further strengthen and topographically tighten as seizures progress and terminate. These findings may have practical clinical implications for targeting specific connections with implantable, therapeutic devices to control seizures.

## Introduction

Localization-related epilepsy causes seizures that arise from one or more abnormal islands of cortical tissue in the neocortex or mesial temporal structures [1]. In more severe cases, seizures with focal onset secondarily generalize, as pathologic activity spreads across the brain [2]. Localization-related epilepsy represents ≈80% of epilepsy cases and is often resistant to medication [3]. For drug-resistant patients, the only treatment options are implantable devices, or more traditionally resective surgery to remove enough cortical tissue in the epileptic network to decrease seizure frequency, while preserving brain tissue responsible for eloquent function. In surgical cases where discrete lesions associated with seizure onset (‘foci’) are not evident on an MRI, only ≈40% remain seizure-free post-surgery [3]. The modest outcome associated with these procedures has lead investigators to further explore spatial distributions of epileptic activity using multiscale neural signals in ECoG and sub-millimeter *μ*ECoG to more accurately localize where seizures start and how their pathologic activity spreads [4–9]. These approaches have spurred a paradigm shift from localizing just the foci towards informing interventions by mapping structural and functional connectivity of the whole epileptic network.

The notion of an epileptic network stems from the idea that pathologic functional connections and/or disconnections disrupt neural function, producing rhythmic motor activity, altered cognition, or abnormal sensation. Functional connections are time-dependent [10] communication pathways between neural populations that are measured by statistical relationships between electrode sensor (node) time series [11], and that evolve according to brain state to produce behavior. The seizure state was originally considered to be hypersynchronous, or composed predominantly of strong functional connections. In contrast, a significant body of recent work presents compelling evidence that complex changes among strong (synchronized) and weak (desynchronized) network nodes accompany seizure dynamics [12–17]. The state-space of these dynamics are well described at the sensor level using measures of node centrality [18, 19]. However, epileptic network architecture at the basic sub-unit of individual connections is poorly understood, but tremendously powerful for discriminating fine-grain network changes that drive seizure dynamics.

Understanding the interplay between individual functional connections in the epileptic network is critical to answer questions goading clinical epileptologists and translational researchers: Where do seizures start? Can the epileptic network be modulated therapeutically? What can these methods reveal about the underlying neurophysiologic mechanisms? Progress in addressing these questions requires methods to track time-dependent functional connections within the epileptic network and understand their relative strengths and weaknesses, which in network terms are collectively referred to as the network’s *geometric* structure. Such methods would not only shed light on geographical dysfunction of epileptic foci, but also the disruption of normal brain tissue that is recruited during seizure events.

We hypothesize that the epileptic network achieves dysfunction and drives seizure activity by reconfiguring network connections during key network states that are clinically described as seizure generation, propagation, and termination. Our network reconfiguration hypothesis is informed by recent work demonstrating that human brain networks dynamically reorganize prior to changes in behavior [20, 21]. During pathologic events, reconfiguration in epileptic networks may involve a redistribution of metabolic resources between strong and weak connections, supporting distinct network functions [22, 23]. Our results support this hypothesis, demonstrating that the epileptic network can be characterized by hubs of persistent strong connections surrounded by rapidly reconfiguring weak connections that drive seizure processes.

## Results

**Fig 1:**
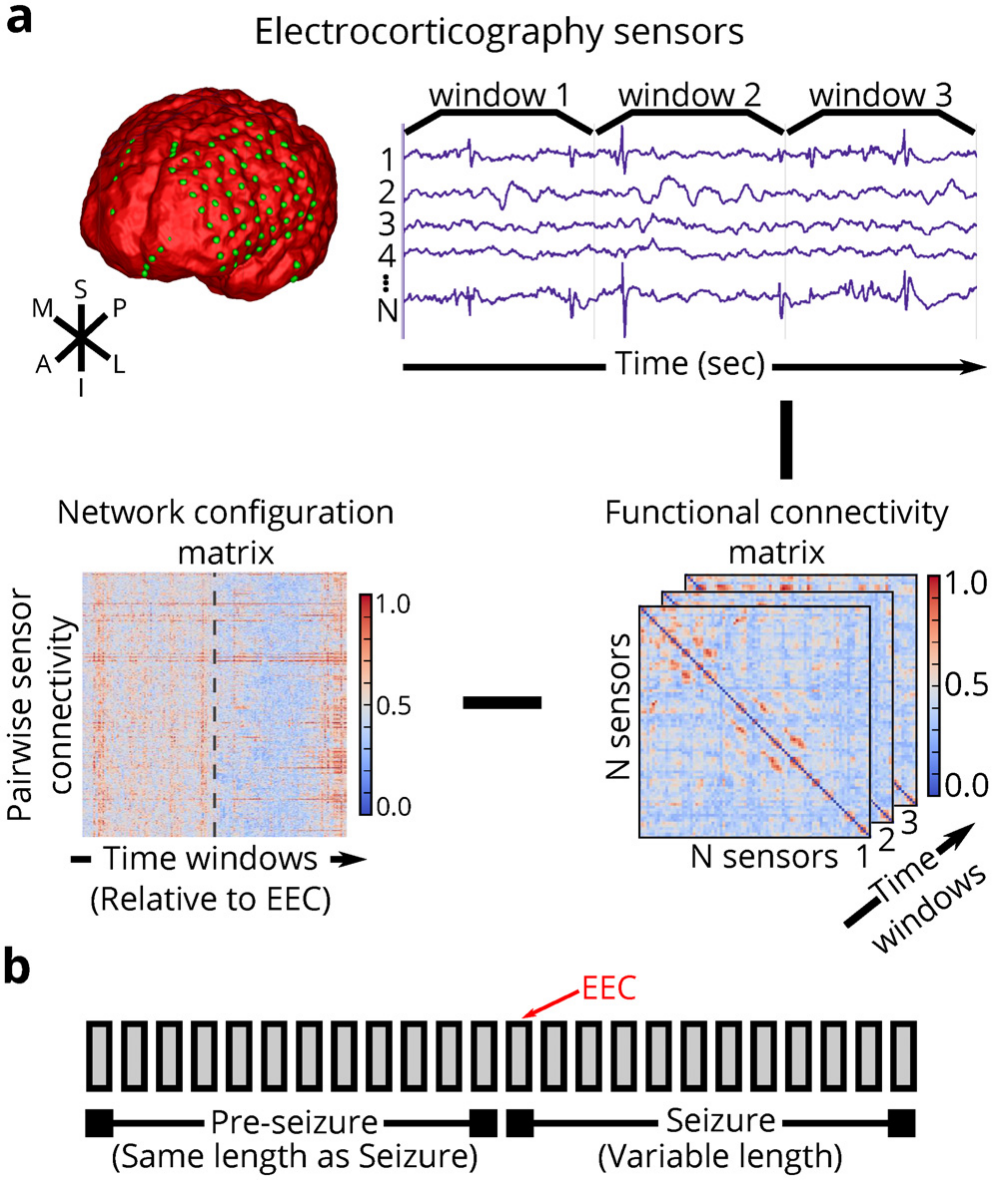
Analysis pipeline for dynamic epileptic networks. (**a**) (***Top***) We create functional networks based on electrophysiology by windowing ECoG signals collected from patients with drug-resistant neocortical epilepsy implanted with intracranial electrodes into 1s time windows. Each sensor is represented as a network node, and weighted functional connectivity between sensors, interpreted as degree of synchrony, is represented as a network connection. (***Lower Right***) Functional connectivity is estimated by a magnitude normalized cross-correlation between sensor time series for each time window. (***Lower Left***) We study temporal dynamics of each unique connection in a network configuration matrix. (**b**) For each epileptic event, we estimate dynamic functional connectivity during the seizure and the pre-seizure epoch. A seizure epoch consists of time windows between seizure onset—as characterized by the earliest electrographic change (EEC) [24]—and seizure termination. The associated pre-seizure epoch consists of an equal number of time windows as the seizure epoch and occurs immediately prior to the EEC.

To analyze the epileptic network, we retrieved ECoG recorded during simple partial, complex partial, and secondarily generalized seizures from 21 neocortical epilepsy patients undergoing routine pre-surgical evaluation of their epilepsy (see Table 1 for patient-specific information) through the *International Epilepsy Electrophysiology Portal* (IEEG Portal, http://www.ieeg.org). We estimated weighted functional connectivity using a normalized cross-correlation metric (see Materials and Methods) applied to non-overlapping, 1s time windows of ECoG (Fig 1a). This procedure results in a symmetric, *N* × *N* *connectivity matrix* (specifying $\frac{N(N-1)}{2}$ unique connections in the upper or lower triangle of the symmetric connectivity matrix), where *N* is the number of network nodes, for each of *T* time windows analyzed. The pattern of unique network connections from a single time window is a *configuration vector*, which can be concatenated over all time windows to form a *configuration matrix* of size $\frac{N(N-1)}{2}\times T$. To better understand how global and local epileptic network geometry drive seizure dynamics, we study the configuration matrix during epileptic events divided into *seizure* and *pre-seizure* epochs (Fig 1b).

**Table 1:** Patient information. Patient data sets accessed through IEEG Portal (http://www.ieeg.org). Age at first reported onset and at phase II monitoring. Localization of seizure onset and etiology is clinically-determined through medical history, imaging, and long-term invasive monitoring. Seizure types are SP (simple-partial), CP (complex-partial), CP+GTC (complex-partial with secondary generalization), or GA (generalized atonic). Counted seizures were recorded in the epilepsy monitoring unit. Clinical imaging analysis concludes L, Lesion; NL, non-lesion. Surgical outcome was based on either Engel score or ILAE score (scale: I-IV/V, seizure freedom to no improvement; NR, no resection; NF, no follow-up). M, male; F, female.

Patient (IEEG Portal)	Sex	Age (Years) (Onset/Surgery)	Seizure Onset	Etiology	Seizure Type	Seizures (N)	Imaging	Outcome
HUP64_phaseII	M	03/20	Left frontal	Dysplasia	CP+GTC	01	L	ENGEL-I
HUP65_phaseII	M	02/36	Right temporal	Auditory reflex	CP+GTC	03	N/A	ENGEL-I
HUP68_phaseII	F	15/26	Right temporal	Meningitis	CP, CP+GTC	05	NL	ENGEL-I
HUP70_phaseII	M	10/32	Left perirolandic	Cryptogenic	SP	08	L	NR
HUP72_phaseII	F	11/27	Bilateral left	Mesial temporal sclerosis	CP+GTC	01	L	NR
HUP73_phaseII	M	11/39	Anterior right frontal	Meningitis	CP+GTC	05	NL	ENGEL-I
HUP78_phaseII	M	00/54	Anterior left temporal	Traumatic injury	CP	05	L	ENGEL-III
HUP79_phaseII	F	11/39	Occipital	Meningitis	CP	01	L	NR
HUP86_phaseII	F	18/25	Left temporal	Cryptogenic	CP+GTC	02	NL	ENGEL-II
HUP87_phaseII	M	21/24	Frontal	Meningitis	CP	02	L	ENGEL-I
Study 004-2	F	14/27	Right temporal occipital	Unknown	CP+GTC	01	NL	ILAE-IV
Study 006	M	22/25	Left frontal	Unknown	CP	02	NL	NR
Study 010	F	00/13	Left frontal	Unknown	CP	02	L	NF
Study 016	F	05/36	Right temporal orbitofrontal	Unknown	CP+GTC	03	NL	ILAE-IV
Study 019	F	31/33	Left temporal	Unknown	CP+GTC	15	NL	ILAE-V
Study 020	M	05/10	Right frontal	Unknown	CP+GTC	04	NL	ILAE-IV
Study 023	M	01/16	Left occipital	Unknown	CP	04	L	ILAE-I
Study 026	M	09/09	Left frontal	Unknown	CP	10	NL	ILAE-I
Study 031	M	05/05	Right frontal	Unknown	CP+GTC	05	NL	NF
Study 033	M	00/03	Left frontal	Unknown	GA	07	L	ILAE-V
Study 037	F	45/??	Indeterminate	Unknown	CP	02	NL	NR

### Epileptic Network Reconfiguration Reveals Distinct Seizure States

**Fig 2:**
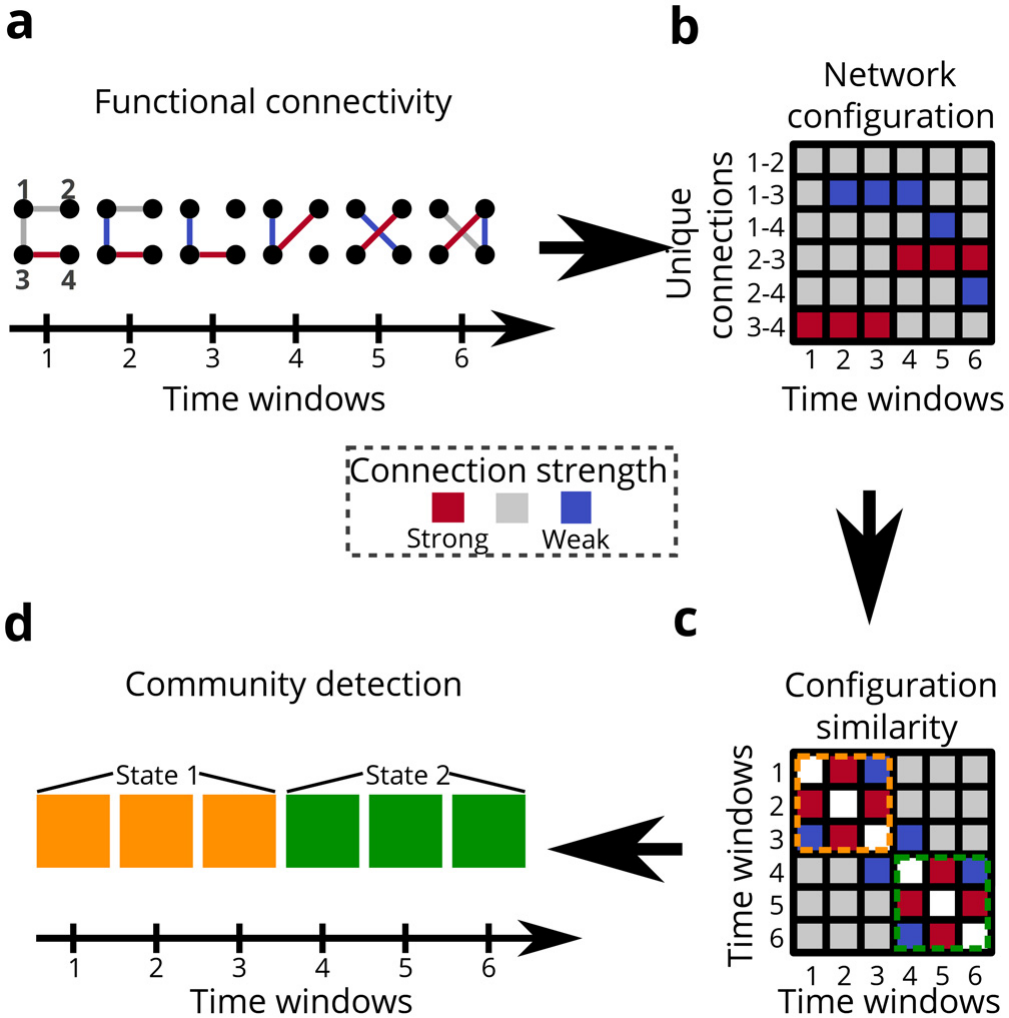
Schematic for identifying network configuration states. (**a**) We estimate dynamic functional connectivity; colors represent arbitrary connection strengths ranging from strong to weak (red, gray, blue). (**b**) We track all unique functional connections over time using a configuration matrix, in which each vector represents the set of connection weights for a 1s time window. (**c**) We compute the similarity between the network geometries of each pair of time windows using a Pearson correlation coefficient. In the resultant configuration-similarity matrix, colors represent the magnitude of similarity and visually identified clusters are distinguished by colored, dashed lines (orange and green). (**d**) We optimize a modularity quality function to cluster the configuration vectors (and thus time windows) into communities. Each cluster or community contains time windows with similar network geometry; colors represent assignments of time windows to different network configuration communities (orange and green).

Do functional connectivity patterns significantly change as a seizure progresses? To answer this question, we developed a new method to uncover network states, defined by unique patterns of sensor-sensor functional connectivity between *T* time windows (Fig 2). We define a *network state* to be the set of all configuration vectors that exhibit a similar pattern of functional connectivity, more formally known in the network science literature as “network geometry”. To quantify geometric similarity, we calculated the Pearson correlation coefficient between configuration vectors extracted from all possible pairs of *T* time windows. This procedure produced a symmetric *T* × *T* configuration-similarity matrix (Fig 2c).

**Fig 3:**
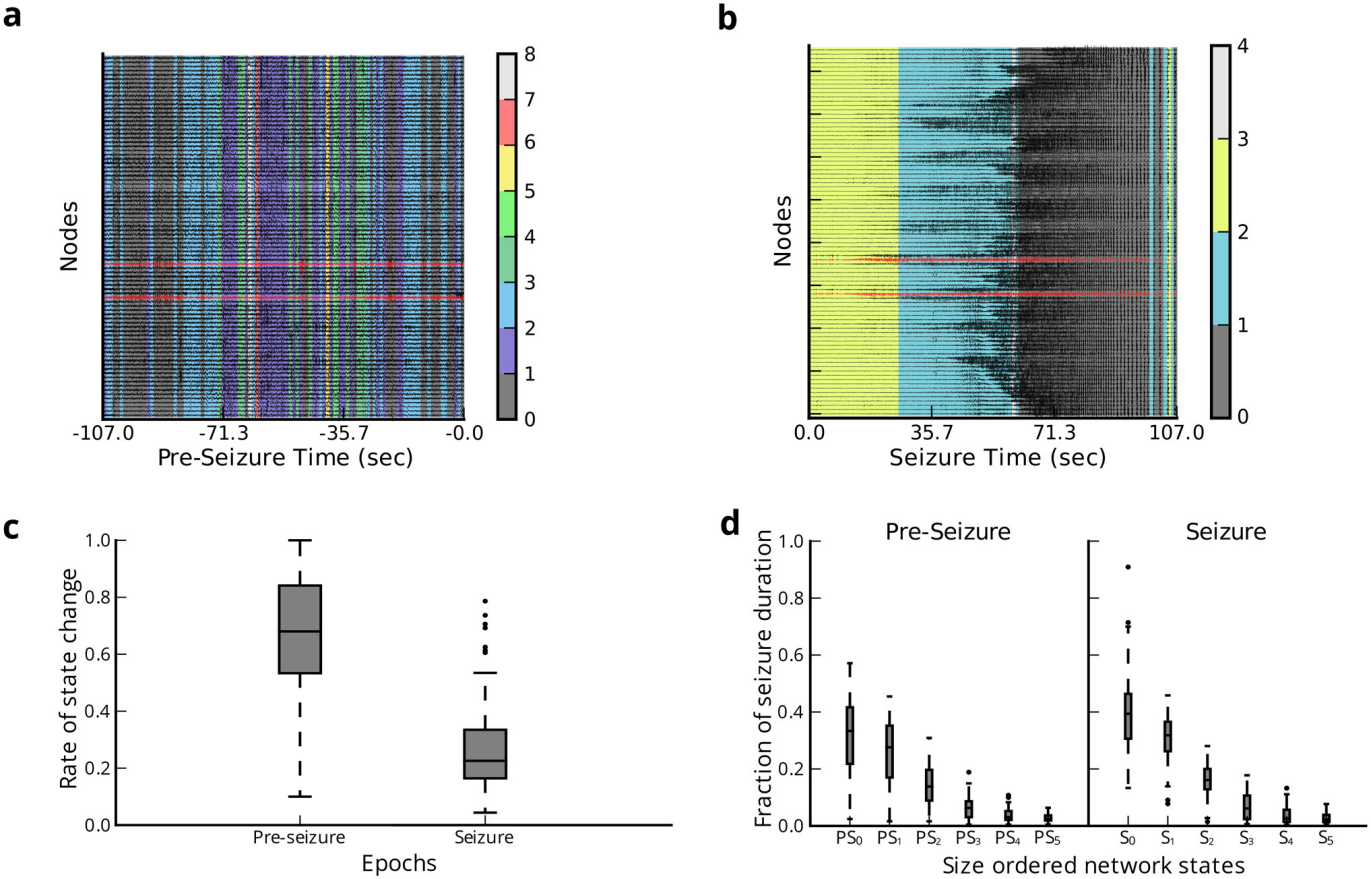
Distinct dynamical states of epileptic networks. (**a**) Example clustering of time windows to network states during a single pre-seizure epoch demonstrating rapid network reconfiguration. State assignments are overlaid on ECoG signals. Red traces correspond to seizure onset nodes. (**b**) Example clustering of time windows to network states during associated seizure epoch (from EEC to Termination) demonstrating slower network reconfiguration. (**c**) Network flexibility—or average rate of network state transitions—during pre-seizure and seizure epochs. The epileptic network displayed significantly more geometric reconfigurations during pre-seizure epochs than in seizure epochs (*N* = 88, *p* = 2.2 × 10^−16^). (**d**) Size-ordered distribution of total fractional duration of the 6 longest network states from each epoch (*PS* (*S*) indicates states of pre-seizure (seizure) epochs). All epochs are normalized to have duration of 1. We retain the first 3 network states of each epoch (*PS*_0_, *PS*_1_, *PS*_2_, *S*_0_, *S*_1_, *S*_2_) for the remaining analysis.

We next ask whether clusters of time windows exhibit similar configuration patterns indicative of independent network states (Fig 2d). To test for distinct states in each epileptic event, we used an unsupervised clustering approach for networked data—community detection—that maximizes a modularity quality function **Q** obtained from the configuration-similarity matrix (see Materials). In this approach, a structural resolution parameter *γ* can be tuned to maximize the reliability of state estimates; we separately tuned this parameter for each seizure and pre-seizure epoch in each patient (see S1 Text). This procedure assigns each time window to a community (or *state*), and each state is composed of time windows that exhibit similar network geometry. Note that these time windows need not be temporally contiguous. We found that the epileptic network transitions through a variety of network states during pre-seizure and seizure epochs (Fig 3a–3b). A comprehensive summary of epoch and state durations for each patient can be found in **Table A** in S1 Text.

The existence of epileptic state transitions support the notion of a dynamically reconfiguring network. To quantify reconfigurability of the epileptic network, we measured the network *flexibility*, or rate the of state change in each epoch (Fig 3c). We found that pre-seizure epochs display significantly higher flexibility (*μ* = 0.665±0.205) than seizure epochs (*μ* = 0.274±0.165) (paired-samples *t*-test; *t*_87_ = −14.12, *p* = 2.2 × 10^−16^), indicating that the epileptic network transitions between states more slowly through seizure epochs than through pre-seizure epochs. Furthermore, pre-seizure epochs consisted of many short-duration states, while seizure epochs consisted primarily of 3 long states that occupy ≈87% of seizure duration (Fig 3d). The three largest pre-seizure states occupied approximately 75% of the epoch. Together, these results support the possibility that rapid changes in network geometry in pre-seizure epochs lead to seizures, and once there, the network undergoes slower geometric changes through 3 main dynamic states. To fairly assess differences in seizure and pre-seizure states, we retained the 3 longest network states from seizure (*S*_0_, *S*_1_, *S*_2_) and pre-seizure epochs (*PS*_0_, *PS*_1_, *PS*_2_) for the following analyses.

### Epileptic Network Redistributes Connectivity during Seizures

**Fig 4:**
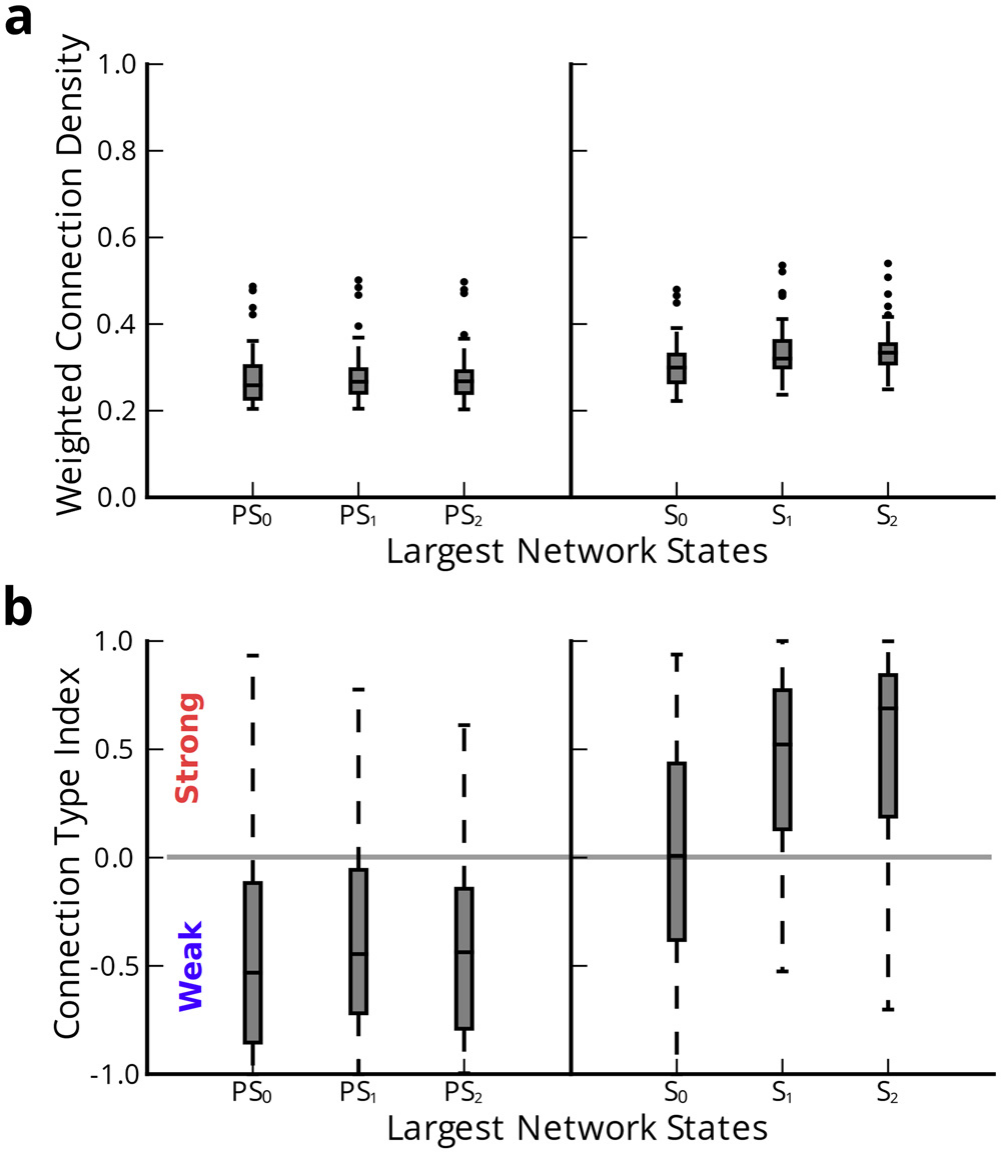
Changes in global connectivity of epileptic networks. (**a**) Functional connection density during pre-seizure (*PS*) and seizure (*S*) network states. We average connection strengths over all time windows within each network state (*N* = 80). We found significant increase in connection density from all *PS* to any *S* network state, and significantly greater connection density during *S*_2_ and *S*_1_ compared to *S*_0_. (**b**) Connection type index indicating strong or weak connection dominance during *PS* and *S* network states (*N* = 80). We found significant change from weak type dominance during *PS* to quasi weak-strong type dominance during *S*_0_ and strong type dominance during *S*_1_ and *S*_2_.

In the previous section, we observed that seizures progress through distinct states characterized by different functional connectivity patterns. To understand how these patterns differ, we used a two-pronged approach, examining (i) the strength of functional connections and (ii) the pattern of functional connections in different network states (Fig 4a). For simplicity, we report the strength of functional connections as a fraction of the total strength, and we refer to this quantity as the *connection density*. Similarly, to characterize the pattern of functional connections, we examine the relative prevalence of synchronized (strong) versus desynchronized (weak) connections, and we refer to this quantity as the *connection type index*.

The functional connection density measures the average connection strength in the network, where greater connection density indicates increased global network synchrony. We computed connection density by averaging the distribution of all connection strengths over all time windows in the given network state. We performed a one-way ANOVA to compare the effect of pre-seizure and seizure network states on connection density. We observed a significant effect of network state on connection density (*F*_5,474_ = 21.34, *p* < 2 × 10^−16^). Post-hoc analysis using Tukey’s honest significant difference test (HSD) to control for a family-wise rejection error rate of 5% (FWER = 5%) revealed a significant increase of connection density in each seizure state compared to any pre-seizure state. During the seizure, connection density increased between *S*_0_ (*μ* = 0.304±0.051) and *S*_1_ (*μ* = 0.333±0.58) (*p*_*adj*_ = 0.014), and *S*_0_ and *S*_2_ (*μ* = 0.338±0.052) (*p*_*adj*_ = 0.002), but did not significantly change between *S*_1_ and *S*_2_ (*p*_*adj*_ = 0.995). Differences in connection density between the pre-seizure states (*PS*_0_, *PS*_1_, *PS*_2_) were not significant. These results suggest synchronization increases as the network transitions from pre-seizure to seizure states.

While we observed an increase in global synchrony as seizures begin and progress, it is unclear whether this increase accompanies a change in functional connectivity pattern, and particularly in a switch from relative desynchronization (weak connectivity) to synchronization (strong connectivity). To type individual connections as strong or weak, we (1) compiled a distribution of all functional connections over all time windows across each event (encompassing the pre-seizure and seizure epoch), and (2) determined thresholds for connection type based on rank percentile, where *strong* (*weak*) connections were stronger (weaker) than 95% of all connections. Based on connection type assignments in each epoch, we found the total number of strong (*C*_*s*_) and weak (*C*_*w*_) connections over all time windows in each network state and computed the connection type index as $\frac{C_{s}-C_{w}}{C_{s}+C_{w}}$. A strong type dominant network has a connection type index between 0 and +1, where +1 implies all connections are strong, while a weak type dominant network has a connection type index between 0 and −1, where −1 implies all connections are weak.

To determine the effect of network state on connection type index (Fig 4b), we conducted a one-way ANOVA test. We observed a significant effect of network state on connection type index (*F*_5,474_ = 70.41, *p* < 2 × 10^−16^). Post-hoc analysis using Tukey’s HSD (FWER = 5%) indicated a significant change from weak type dominance during any pre-seizure state towards strong type dominance during seizure states. During the seizure, connection type index increased between *S*_0_ (*μ* = 0.023±0.498) and *S*_1_ (*μ* = 0.437±0.417) (*p*_*adj*_ < 2 × 10^−16^), and *S*_0_ and *S*_2_ (*μ* = 0.512±0.447) (*p*_*adj*_ < 2 × 10^−16^), but did not significantly change between *S*_1_ and *S*_2_. Differences of connection type index between the pre-seizure states (*PS*_0_, *PS*_1_, *PS*_2_) (*μ* ≈ −0.401) were not significant.

Chronologically, the network is persistently desynchronized during the pre-seizure epoch, is driven to a quasi-synchronized seizure generation state *S*_0_, and remains persistently synchronized as the seizure progresses through *S*_1_ and *S*_2_. A predominance of weak connections during a persistently desynchronized pre-seizure epoch coincides with earlier findings of improved network flexibility to reorganize during the same epoch. Unremarkable change in weak connection type dominance during the pre-seizure epoch suggests that the network simply redistributes weak connections amongst different nodes during this period. A critical transition to seizure generation during state *S*_0_ is accompanied by synchronization towards more evenly distributed strong and weak connection types. As network flexibility decreases during the seizure, connections become more strong type dominant. To better understand how the network evolves through the desynchronized and synchronized states, we next study the impact of local, geographical changes in network geometry.

### Dynamic Regional Structure of the Epileptic Network

**Fig 5:**
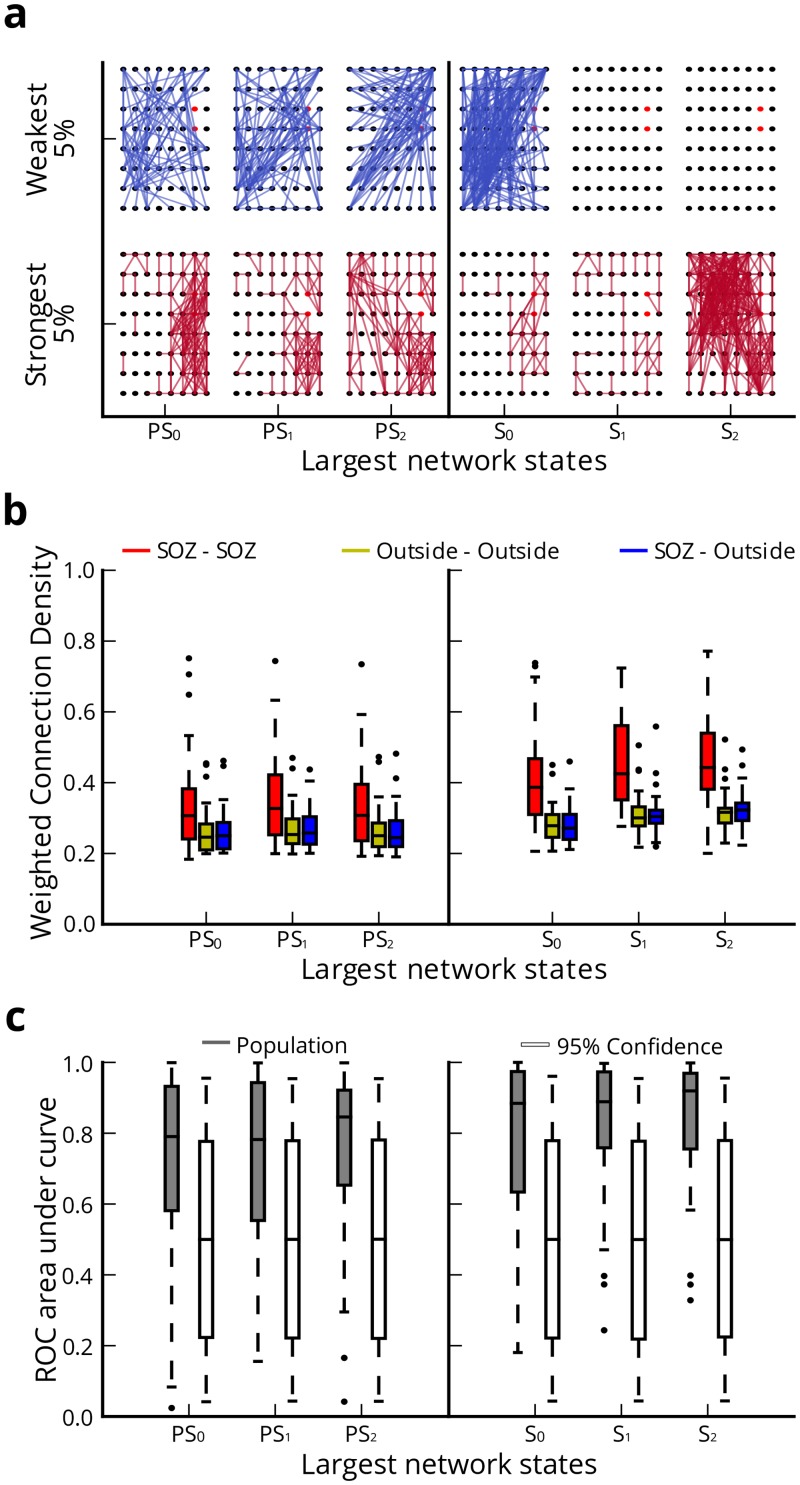
Regional characteristics of network geography. (**a**) Example of network geography with preserved 2-D spatial relationships between nodes for categories of strongest and weakest connections in upper and lower 5% of connection strength distribution; Connection colors indicate weak (blue) and strong (red); the clinically-determined seizure onset sensors are shown in red. (**b**) Connection strength within 3 geographic connection types during *PS* and *S* network states (*N* = 50). During *PS* and *S*, we observed significantly stronger connections amongst SOZ-SOZ regions than OUT-OUT and SOZ-OUT regions. During *S*, we observed significant increase in SOZ-SOZ connections as seizures initiate and progress. (**c**) ROC AUC compared to 95% bootstrapped confidence intervals using connection strength to predict SOZ-SOZ connections during *PS* and *S* network states (*N* = 50). The synchronized *S*_2_ state yielded the best performance, while the desynchronized *PS*_0_ state yielded the worst performance.

In the preceding analyses, we demonstrated that the epileptic network displays weak type dominant connectivity during pre-seizure epochs and undergoes synchronizes to strong type dominance as the seizure initiates and progresses through 3 primary states. However, our approaches did not address whether these reconfigurations are spatially localized or distributed, and how they relate to seizure foci. To address these questions, we leveraged routine clinical procedures: A team of neurologists successfully identified the sensors on the seizure onset zone (SOZ) based on visual inspection of the intracranial recordings in 15 patients across a total of 50 seizures. We used this information to map connections in each seizure state to physical electrode locations in stereotaxic space (Fig 5a).

To quantify spatial localization of connectivity relative to seizure foci and examine the role of network region in pre-seizure and seizure dynamics, we delineated the following three geographic types: (i) connections between nodes within the SOZ (SOZ-SOZ), (ii) connections between nodes outside the SOZ (OUT-OUT), and (iii) connections between one node within the SOZ and one node outside the SOZ (SOZ-OUT) (Fig 5b). We performed a two-way ANOVA test to compare the effects of geography and network state on connection strength. We observed a significant main effect of geography on connection strength (*F*_2,882_ = 158.501, *p* < 2 × 10^−16^) and a significant main effect of network state on connection strength (*F*_5,882_ = 26.394, *p* < 2 × 10^−16^). We also observed significant interactions between geography and network state (*F*_10,882_ = 2.871, *p* = 0.002). Post-hoc analysis on the interactions using Tukey’s HSD (FWER = 5%) identified persistently stronger connection strength amongst SOZ-SOZ connections (*μ* ≈ 0.393±0.140) relative to OUT-OUT (*μ* ≈ 0.282±0.059) and SOZ-OUT (*μ* ≈ 0.284±0.059) connections in every network state (*p*_*adj*_ < 1 × 10^−3^). Connections in the SOZ-SOZ group were modestly strengthened during *S*_0_ relative to *PS*_0_ and *PS*_2_ (*p*_*adj*_ < 0.05), were greatly strengthened during *S*_1_ and *S*_2_ relative to any pre-seizure state (*p*_*adj*_ < 1 × 10^−3^), and during the seizure only strengthened between *S*_0_ to *S*_2_ (*p*_*adj*_ < 0.05). However, connection strengths in the SOZ-SOZ group did not significantly vary between pre-seizure states. Similarly, SOZ-OUT and OUT-OUT group did not significantly vary between any network states.

These results suggest that SOZ-SOZ connections are persistently the strongest of all network connection types during pre-seizure and seizure epochs. Upon seizure generation SOZ-SOZ connections strengthen incrementally, and then substantially as seizures progress. Nuancing our description of global network connectivity during pre-seizure and seizure epochs, which demonstrates a progression from desynchronization to synchronization over time, our results demonstrate that (i) desynchronization during pre-seizure states is primarily localized to SOZ-OUT and OUT-OUT connections, and (ii) resynchronization is primarily localized to SOZ-SOZ connections. Intuitively, desynchronous SOZ-OUT and OUT-OUT connections that frequently re-wire drives heightened network flexibility during pre-seizure epochs and synchronous SOZ-SOZ connections disrupts network flexibility during the seizure.

To investigate the sensitivity and specificity of connection strength as a measure for identifying SOZ-SOZ connections, we employed receiver operating characteristic (ROC) analysis during pre-seizure and seizure epochs (Fig 5c). The ROC analysis evaluates the sensitivity and specificity of connections belonging to the SOZ-SOZ type as connection strength threshold is incrementally raised. We evaluate performance in detecting SOZ-SOZ connections by computing the area under the ROC curve (AUC) ranging from 0 to +1, where values of +1 imply low sensitivity and false positives with high specificity and true positives. To assess significance of the AUC, we bootstrapped confidence intervals (*α* = 0.05) by re-assigning sensors to the SOZ uniformly at random without replacement 10000 times for each network state in both epochs. During seizure epochs, we found that *S*_2_ was most effective at predicting SOZ-SOZ connections based on AUC (*μ* = 0.849±0.169) with significant AUC values in 32 of 50 seizures. Conversely *S*_0_ was least effective at predicting SOZ-SOZ connections (*μ* = 0.773±0.238) with significant AUC values across 26 of 50 seizures. During pre-seizure epochs, SOZ-SOZ connections were similarly predictable across *PS*_0_ (*μ* = 0.709±0.268) (significant in 25 of 50), *PS*_1_ (*μ* = 0.722±0.257) (significant in 25 of 50), and *PS*_2_ (*μ* = 0.754±0.238) (significant in 27 of 50). These results suggest that connection strength may be used to predict SOZ-SOZ connections during pre-seizure epochs with precision, but has better performance during more synchronized states such as *S*_2_ compared to less synchronized states such as *S*_0_.

### Impact of Surrounding Connectivity on Epileptic Network Dynamics

Thus far we have seen how connectivity associated with the SOZ synchronizes the epileptic network during seizures. However, it is unclear whether involvement from the broader epileptic network aids or disrupts pre-seizure and seizure dynamics.

**Fig 6:**
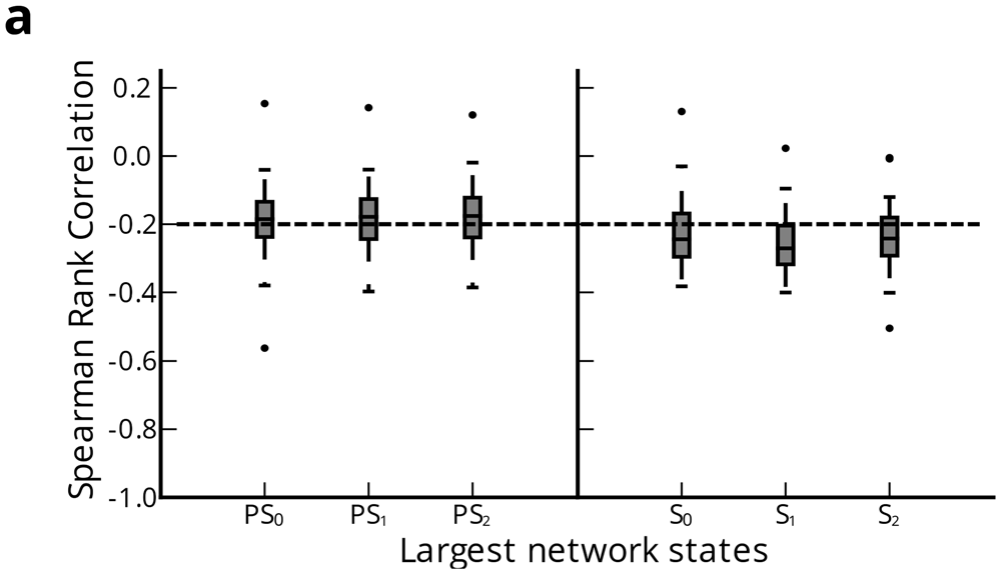
Topographical characteristics of network connectivity. (**a**) Average Spearman’s rank correlation coefficient between connection length and strength in *PS* and *S* epochs (*N* = 75). Physical connection lengths computed from regularly spaced nodes in electrode grid; connection lengths measured in millimeters. Stronger connections are consistently shorter than weaker connections. During *S*, there is significant topographical reorganization making weak connections longer and strong connections tighter.

We first hypothesized that changes in network geometry are not limited to redistribution of connection strengths, but may also involve topographical changes in connection lengths accompanying changes in functional network anatomy. In a sample of pre-seizure and seizure states, we observed clustering of strong connections while weak connections distributed more broadly (Fig 5a). To test our hypothesis, we restricted our analysis to connections within electrode grids with uniformly spaced nodes in 8 × 8, 8 × 6, 6 × 6, or 4 × 6 configurations (in 75 seizures over 19 patients) and computed average Spearman’s rank correlation coefficient between connection length and connection strength over all time windows of each network state (Fig 6a). A more positive (negative) correlation coefficient indicated stronger connections were longer (shorter). A one-way ANOVA test was conducted to compare the effect of pre-seizure and seizure network states on correlation between connection length and connection strength. We observed a significant effect of network state on correlation (*F*_5,444_ = 9.348, *p* = 1.76 × 10^−8^). Post-hoc analysis using Tukey’s honest significant difference test (HSD) to control for a family-wise rejection error rate of 5% (FWER = 5%) revealed significant increase in negative correlation between connection length and strength in *S*_0_ (*μ* = −0.225±0.92) compared to *PS*_2_ (*μ* = −0.182±0.091) (*p*_*adj*_ < 0.05) but not *PS*_0_ (*μ* = −0.188±0.100) or *PS*_1_ (*μ* = −0.189±0.092). Connection length is significantly more negatively correlated with connection strength in *S*_1_ (*μ* = −0.258±0.081) and *S*_2_ (*μ* = −0.242±0.086) compared to any pre-seizure state (*p*_*adj*_ < 0.01). There was no significant change in correlation between pre-seizure states or seizure states.

In summary, we found that stronger connection strengths are present in connections with shorter lengths, regardless of network state. During seizures, reorganization in the epileptic network leads to further lengthening of weaker connections and shortening of stronger connections. Coinciding with the earlier finding that seizure generation involves quasi-synchronization of the network, we find a modest shortening of strong connections relative to the pre-seizure period. As seizures progress, synchronous connections tighten to more local regions, while desynchronous connections stretch further into the broader epileptic network.

## Discussion

### Epileptic Network Reconfiguration

Intuitively, complex reconfiguration of functional brain networks can accompany changes in cognitive state or changes in behavior. Prior fMRI studies have explored such reconfiguration in whole-brain networks constructed from data acquired during motor skill learning [20] and as task states change [25], and in networks impacted by stroke [26, 27]. In contrast, here we explore the reconfiguration of a local area and use higher resolution ECoG data to map the fine-scale temporal dynamics of reconfiguration processes.

In this study, we developed and exercised a novel method for distinguishing brain states based on differences in time-dependent functional network geometry. Our approach expands upon previous notions of state-space in dynamic epileptic networks [18, 19], by tracking changes between node pairs (connections) rather than in node importance (centrality). An important advantage associated with this technique is that network reorganization can be studied without *a priori* knowledge of specific topological structure, such as small-worldness [16]. Rather, time-dependent changes in connectivity are based simply on similarities in signal statistics.

We applied our technique to a set of human ECoG recordings, and extracted network dynamics during seizure and pre-seizure epochs. We found that seizures exhibit at least three network states (*S*_0_, *S*_1_, *S*_2_) and that the epileptic network progresses through these states more slowly in comparison to the period preceding seizure generation. Our results are in line with prior work that has shown more frequent state changes during the interictal period in comparison to seizures [19]. Next, we provide a mechanistic explanation of how state changes operate with strong and weak regimes of connectivity to drive seizures through neurologically-defined onset, propagation and termination states ubiquitous in clinical descriptions.

### Balance of Strong and Weak Connections

Our analytical approach utilizes the distribution of functional connection strengths to characterize connections as “strong” (synchronous) or “weak” (desynchronous), rather than simply stating that two sensors are functionally “connected” or “not connected”. Mathematically, this focus corresponds to a study of network *geometry* as opposed to network *topology*. A primary advantage of the weighted network approach is the ability to separate connections into classes that differ in strength. Evidence suggests that strong and weak connections play different roles in supporting cognitive function [23, 28]. Traditional thought is that strong connections represent primary communication pathways between brain areas. However, recent work demonstrates that weak connections support increased network efficiency and may play a large role in distinguishing pathologic [29] and healthy [23, 30] network states. From a dynamical perspective, strong connections may persistently engage throughout neurophysiological processes, whereas weak connections may engage transiently to enable brain state transitions.

Prior work has speculated that the epileptic network is connected at the beginning of the seizure, disconnected in the middle, and finally reconnected at the end [12, 14, 16]. However, our results suggest that a more accurate way to address this hypothesis is to consider the strength of functional connections and disambiguate slower temporal dynamics occurring at each node, independently, which may elevate spurious connectivity between disconnected regions.

Using a weighted connectivity approach, we find that connections in the epileptic network have more weak than strong connections during *PS*_0_, *PS*_1_, and *PS*_2_, states preceding the electrographic seizure onset, a near balance of strong and weak connections during *S*_0_, which corresponds to seizure generation, and more strong than weak connections during *S*_1_ and *S*_2_ states representing seizure progression and termination. It is possible that clinician subjectivity in marking the time of seizure onset may explain our result of disconnectivity before seizure generation, which contrasts with prior reports of a disconnected network at either seizure onset or mid-seizure [12, 14, 16]. Our method places greater emphasis on connectivity derived from faster activity by reducing contribution from slower dynamics (see Materials and Methods) and corroborates clinical belief that seizure generation during *S*_0_ involves a gradual transition from desynchronous to synchronous connectivity, which peaks during the termination phase of the seizure (*S*_2_).

Mechanistically, the weak connectivity that we observe preceding seizure generation benefits from high network flexibility to drive seizure generation through a rapid reorganization of weaker connections in the epileptic network. As seizures initiate and progress, the epileptic network redistributes weak connectivity to strong connectivity while network flexibility is concurrently diminished. In relation to prior work that demonstrates a propensity for the epileptic network to follow a recurring pattern of state transitions during seizures [19], our results suggest that weak connectivity preceding the seizure drives the network to a more predictable series of increasingly synchronized states during seizures. Next, we explore beyond global network structure and discuss how regional connectivity dynamics provide further insight on network drivers of seizure evolution.

### Regional Connectivity Regulates Seizure Evolution Dynamics

While temporal network structure provides rich information regarding seizure states, it does not directly provide information regarding the spatial processes involved in seizure dynamics. We therefore complemented the temporal network approach by incorporating information about sensor role either within or outside the seizure onset zone and sensor location in Euclidean space. Our results demonstrate that these additional spatial features provide new insights into potential neurophysiological mechanisms involved in seizure generation, and may inform the development of clinical tools for objectively isolating the seizure onset zone directly from seizure or pre-seizure data.

Prior work has demonstrated high synchronization within the seizure onset zone during interictal epochs [31, 32]. However, the temporal dynamics and geometrical roles of these two sets of areas has remained elusive. Our results elucidate the role played by seizure onset regions during seizures and the accompanying recruitment of the surrounding epileptic network during termination. Clear isolation of the seizure onset zone exists in pre-seizure periods, suggesting the potential to identify foci, niduses of seizure generation, within the network from inter-ictal data. Critically, connectivity within the onset zone strengthens during early seizure periods (*S*_1_) and intensifies as seizures progress (*S*_2_) and terminate (*S*_3_), suggesting that the onset zone drives the transition from global desynchronization to synchronization during seizure generation and persists in this functional role through the entire seizure. Such a mechanism also points to a role of the onset zone in seizure termination, potentially in tandem with topographical mechanisms, which we discuss in the next section.

### Network Tightening during Seizures

Our observation that stronger connections are typically short and weaker connections are typically long, is consistent with results from two lines of research: (i) functional studies in healthy individuals that utilize other imaging modalities such as fMRI [23] and (ii) structural connectivity studies in non-human primates that utilize tract tracing techniques [22]. In epilepsy, prior work has shown hubs of connectivity proximal to the seizure onset zone [18, 33, 34], however their role in seizure dynamics was previously unknown. We show that seizure generation leads to further shortening of stronger connections and lengthening of weaker connections, suggesting that stronger connections are physically tightening, perhaps into more functionally cohesive portions of cortex during seizures. We speculate that the tightening of stronger connections to localized sub-networks might act as a control mechanism to quench disruptive network activity that may have built-up over many hours prior to the seizure through increasing frequency of epileptiform discharges [24] or facilitate previously described compensatory mechanisms [18]. Conversely, weaker connections are longer during seizure periods than pre-seizure periods and could be a vehicle for spreading desynchronous activity broadly.

### Clinical Impact and Future Work

We have seen that the dynamical processes that propel epileptogenic networks into seizures can be complex and are poorly understood. Yet, clinicians rely on visual inspection to describe spatial and temporal properties of seizures. The lack of standardized clinical measures to mark epileptic events calls for the development of automated methods. The network analysis tools we have built, while generally applicable to any dynamic network, can parse seizure states, localize driver ‘foci’ of seizures, and characterize how seizures progress and terminate. This interpretation can be translated into useful clinical tools to identify dysfunctional anatomical regions that drive the epileptic network and may be particularly amenable to local interventions, such as surgery or device placement. Of interest, seizure driving ‘foci’ were equally present in the half of our study patients who did not have focal lesions on brain imaging, compared to those patients with lesions demonstrated on MRI. We plan a more detailed study in the future to correlate mapping of these seizure-driving regions with brain resection and outcome.

Currently, our tools employ community detection techniques to identify gross changes in the meso-scale architecture of network structure across time. The observed meso-scale reconfiguration processes may be accompanied by region-specific trends in reconfiguration between the epileptic network and surrounding healthy networks. A remaining gap is understanding how functional dynamics map to structural features of the epileptic network using fiber-tracking techniques to describe how seizures start and then spread through white-matter. Additionally, this work could be used to address cellular mechanisms by considering micro-scale reconfigurations. Recent studies suggest that epileptic networks in the neocortex may be composed of distributed micro-domains on the scale of a few cortical columns generating high frequency oscillations and micro-seizures that coalesce in a network during seizure generation and termination [6]. While of great interest, these studies are currently limited by the lack of appropriate implantable high-resolution sensors capable of covering clinically relevant areas sufficiently to yield comprehensive high-resolution maps. Further development of dynamic community detection methods to identify and track reconfiguration within network sub-regions at both the meso and micro-scales may help delineate healthy and pathologic networks and uncover mechanisms of network recruitment.

An important clinical consideration related to this work is the impact of sampling error inherent in any intracranial implantation procedure on our results. Any technique used to map epileptic networks, subdural electrode strips and grids, more distributed “Stereo EEG” implantations, and combinations of these two approaches, usually yield incomplete representations of epileptic networks. It is not possible to fully record from the entirety of cortex in affected patients. In some cases this might mean that neither seizure onset zones nor all regions of seizure spread are fully delineated. Despite this incomplete representation, the presence of three clear states defining seizures in each of the patients presented above, and their objective and independently determined relationship to the seizure onset zone suggest that our findings are important and real. With further validation on a larger number of patients with both lesional and non-lesional epilepsies, we hope to demonstrate the utility of our method to define functional components of epileptic networks. Our method shows promise for informing epilepsy surgery and for placing devices into regions that drive seizure generation and termination. Future work will focus on using these methods to compare competing approaches for localizing epileptic networks, such as subdural and stereo EEG. It is intuitively plausible that each will have advantages in recording components of epileptic networks in different types of localization-related epilepsy.

## Materials and Methods

### Ethics Statement

All patients included in this study gave written informed consent in accord with the University of Pennsylvania Institutional Review Board and Mayo Clinic Institutional Review Board for inclusion in this study.

### Patient Data Sets

Twenty-one patients undergoing surgical treatment for medically refractory epilepsy believed to be of neocortical origin underwent implantation of subdural electrodes to localize the seizure onset zone after noninvasive monitoring was indeterminate. De-identified patient data was retrieved from the online International Epilepsy Electrophysiology Portal (IEEG Portal) [35]. ECoG signals were recorded and digitized at either 512 Hz (Hospital of the University of Pennsylvania, Philadelphia, PA) or 500 Hz (Mayo Clinic, Rochester, MN) sampling rate. Surface electrode (Ad Tech Medical Instruments, Racine, WI) configurations, determined by a multidisciplinary team of neurologists and neurosurgeons, consisted of linear and two-dimensional arrays (2.3 mm diameter with 10 mm inter-contact spacing) and sampled the neocortex for epileptic foci (depth electrodes were first verified as being outside the seizure onset zone and subsequently discarded from this analysis). Signals were recorded using a referential montage with the reference electrode, chosen by the clinical team, distant to the site of seizure onset and spanned the duration of a patient’s stay in the epilepsy monitoring unit.

### Description of Seizure Events

We analyzed a total of 88 seizure events, including simple partial, complex partial, and secondarily generalized, stemming from neocortical foci in this study. Seizure onset time and localization were defined by the point of earliest electrographic change (EEC) and annotated and marked by a team of practicing epileptologists [24]. ECoG signal directly preceding each seizure and equal in duration to that seizure was also extracted for balanced comparison and labeled as pre-seizure.

### Extracting Dynamic Functional Networks

Signals from each epoch were divided into 1-second, non-overlapping, wide-sense stationary time windows in accord with other studies [16] and subsequently pre-processed. To test the biasing effect of high-amplitude spiking on signal connectivity measurements, we also investigated windows 0.5-seconds in duration to sample more of the non-biasing temporal space and found similar results. In each time window, signals were re-referenced to the common average reference [16, 36] to account for variation in reference location across patients and to avoid broad field effects that may bias connectivity measurements erroneously in the positive direction. Each window was filtered at 60 Hz to remove line-noise, and low-pass and high-pass filtered at 120 Hz and 1 Hz, respectively, to account for noise and drift. To correct for correlated signal dynamics for each individual node, we pre-whiten signals in each window and reduce autocorrelation effects for time lags greater than zero. This accomplishes two goals: (i) flattening of the signal power spectrum to enhance higher-frequency content that contains local neural population dynamics, and (ii) decreases the influence of independent node dynamics when computing correlation-based connectivity measurements [36–39].

Dynamic functional networks were formed by applying a normalized cross-correlation similarity function ***ρ*** between the time series of two sensors in the same time window using the formula

$$\begin{array}{c} \rho_{\text{xy}}\left(k\right)=\left|\;E[\left(x_{k}\left(t\right)-\mu_{x_{k}}\right)\left(y_{k}\left(t+\tau \right)-\mu_{y_{k}}\right)]\;\right| \end{array}$$ (1)

where **x** and **y** are signals from one of **N** sensors or network nodes, **k** is one of **T** non-overlapping, one-second time windows, and **x**_**k**_ = **y**_**k**_ = 0. The **N**x**N**x**T** similarity matrix is also known as a network adjacency matrix **A** (Fig 1a). In our weighted network analysis approach, we retain and analyze all possible connection weights between nodes.

### Computing Network States

Network states, or temporal changes in network geometry, was tracked separately in each epoch by clustering the configuration-similarity matrix through a community detection technique known as modularity optimization. We construct the configuration-similarity matrix by first unraveling **A** to a network evolution matrix $\hat{A}$ describing the weights of $\frac{N(N-1)}{2}$ connections across **T** time windows. Using a Pearson correlation coefficient to measure similarity, we transform $\hat{A}$ to a fully-connected **T**x**T** configuration state adjacency matrix **S**. The configuration adjacency matrix is partitioned into communities by maximizing the modularity index **Q**[40] using a Louvain-like locally greedy algorithm [41]. We employed a Newman-Girvan null model [42, 43] and adaptively determined an optimal structural resolution parameter *γ* per seizure (see S1 Text and [44] for a more detailed discussion of resolution parameters in modularity maximization). We used a consensus partition method with 1000 optimizations per run until we obtained consistent community partitioning [44, 45]. The three longest communities (clusters, or network states) from each seizure were selected for further analysis and re-labeled in order of median temporal occurrence for population-level comparison.

### Distinguishing Connection Types

Connections were classified as *strong* or *weak* based on thresholds determined by the distribution of connection strengths for each epoch separately for each seizure. The strong (weak) connections must be >95% (<5%) of all connection strengths. To measure the dominance of *strong* or *weak* connections, we defined the connection type index as

$$\begin{array}{c} B=\frac{C_{s}-C_{w}}{C_{s}+C_{w}} \end{array}$$ (2)

where **C**_**s**_ and **C**_**w**_ are the average number of strong and weak connections over possible connections and number of time windows.

### Measuring Network Topography

Connection topography metrics were computed for only within-grid electrodes, ignoring all other non-grid electrodes such that inter-electrode spacing in all analyses was kept constant. We related connection strength to the two-dimensional physical distance between nodes (electrode sensors) of that connection in millimeters.

## Supporting Information

S1 Text Contains detailed discussion, methods, and model optimization and robustness. (PDF)
