# Supplementary material for: Dynamic Network Drivers of Seizure Generation, Propagation and Termination in Human Neocortical Epilepsy
Source: PLoS Comput Biol. 2015 Dec 17;11(12):e1004608. doi: 10.1371/journal.pcbi.1004608 (PMC4682976; doi:10.1371/journal.pcbi.1004608)
Supplement: S1 Text — (PDF) [file pcbi.1004608.s001.pdf]

Supporting Information for Dynamic network drivers of seizure generation, propagation and termination in human epilepsy

Ankit N. Khambhati[1,2], Kathryn A. Davis[2,3], Brian S. Oommen[2,3], Stephanie H. Chen[2,3], Timothy H. Lucas[2,4], Brian Litt[1,2,3], and Danielle S. Bassett[1,2,5]

[1]Department of Bioengineering, University of Pennsylvania, Philadelphia, PA 19104, USA
[2]Penn Center for Neuroengineering and Therapeutics, University of Pennsylvania, Philadelphia, PA 19104, USA
[3]Department of Neurology, Hospital of the University of Pennsylvania, Philadelphia, PA 19104, USA
[4]Department of Neurosurgery, Hospital of the University of Pennsylvania, Philadelphia, PA 19104, USA
[5]Department of Electrical and Systems Engineering, University of Pennsylvania, Philadelphia, PA 19104, USA

1 Network states

1.1 Configuration vector

For dynamic networks there is a lack of approaches to quantify how the pattern of connections between nodes, or the gross network configuration, varies over time. Clusters of time windows in which network configurations look similar can be thought as network states. In networks where N nodes stay constant over all T time windows, we can describe the state of $\frac{N(N-1)}{2}$ possible connections between all nodes in each time window $t = 1$ to T by a *configuration vector* $\vec{v_t} \in \mathbb{R}^{\frac{N(N-1)}{2}}$; collectively forming a *configuration matrix* $\mathbf{V} \in \mathbb{R}^{\frac{N(N-1)}{2} \times T}$. Intuitively, changes in $\vec{v_t}$ between different t can imply dynamical changes in network connectivity.

While dynamic networks commonly experience meso-scale changes in connectivity over time, there is currently no way to determine whether these meso-scale changes contribute to gross reconfiguration of the entire network. Using a similarity function (herein we use Pearson correlation coefficient), we quantify the degree of change in connectivity patterns in $\mathbf{V}$ between all T time windows stored in a symmetric *configuration-similarity matrix* $S \in \mathbb{R}^{T \times T}$. In this work we are interested in clustering time windows that express similar network configuration patterns. To this end, we employ static community detection techniques to group time windows with similar gross network configuration.

2 Modularity optimization for community detection

2.1 Theory

Community detection is a technique often used to study meaningful group structure in complex networks by clustering nodes into 'modules' or 'communities'. A community can be thought of as a set of nodes that are connected among one another more densely than they are to nodes in other communities. A popular way to identify community structure is to optimize a quality function known as modularity $\mathbf{Q}$, which has

been shown to extract meaningful functional components of networks by comparing the real network to a null model [1, 2, 3, 4]. While these approaches have been mainly applied to both binarized and weighted static networks, more recent extensions of the model investigate dynamic communities that group nodes over time. In our work we apply static community detection to the configuration-similarity matrix, viewed as a fully connected graph where each node represents the similarity of the configuration vector $\vec{v_t}$ between time windows t_1 and t_2 where $t_1 = t_2 = 0$, to group gross network configuration patterns into communities.

In general, to perform community detection one begins with a network of N nodes and a given set of connections between those nodes. A static network is then represented using an $N \times N$ adjacency matrix $\mathbf{A}$. The element A_{ij} of the adjacency matrix represents a connection from node i to node j where $i = j = 0$, and its value indicates the weight of that connection. Then, $\mathbf{Q}$ is formally defined as:

$$Q = \sum_{ij} [A_{ij} - \gamma P_{ij}] \delta(g_i, g_j) \,, \tag{1}$$

where node i is assigned to community g_i, node j is assigned to community g_j, the Kronecker delta $\delta(g_i, g_j) = 1$ if $g_i = g_j$ and it equals 0 otherwise, γ is the *structural resolution parameter*, and P_{ij} is the expected weight of the edge connecting node i to node j under a specified null model. The choice $\gamma = 1$ is very common, but it is important to consider multiple values of γ to examine groups at multiple scales (see *SI Section 2.2*). A commonly chosen null model is the Newman-Girvan null model [3, 4, 1, 2] defined as:

$$P_{ij} = \frac{k_i k_j}{2m} \tag{2}$$

where $k_i = \sum_j A_{ij}$ is the strength of node i and $m = \frac{1}{2} \sum_{ij} A_{ij}$ is the average strength over all connections.

Maximizing Q partitions the network into communities such that the total within-community connection weight is maximum relative to the null.

2.2 Structural resolution parameter for community detection

The structural resolution parameter γ of the modularity quality function can be used to identify community structure over different topological or geometric scales [5, 6, 3, 7, 8]. Typically, γ is chosen to be equal to 1. However, by varying γ, one can examine fine grain structure in networks by extracting smaller communities. In our application, we were interested in determining community structure of configuration-similarity matrices with a variable (i) number of sensors implanted per patient, (ii) seizure duration and (iii) spatial extent of epileptogenic cortex. Each of these factors can potentially impact the scale at which appreciable meso-scale functional reorganization can be detected.

Prior studies have explored a structural resolution parameter limit that represents a trade-off between grouping all nodes into a single community with large modularity (low γ) and grouping each node into separate communities with low modularity (high γ) [6]. In real-networks [8] modularity and structural resolution share an approximate inverse power relationship, suggesting an optimal γ may occur as rate of change in modularity decreases (within increasing γ). Such a phenomenon would occur at the inflection point for change in modularity as the γ parameter is tuned.

To determine an optimal γ parameter for each configuration-similarity matrix (per epoch, per patient), we computed the inflection point in modularity $\mathbf{Q}$ at each γ between 0.8 and 1.3 in intervals of 0.01 (**Fig. A**). We define the optimal γ to be the γ at which the inflection point occurs. The optimal γ (**Fig. B**) was significantly different between the pre-seizure epochs and seizure epochs, based on t-test ($t_{87} = 2.64$, $p < 0.01$). This result demonstrates that the granularity of network state division is significantly greater during seizure epochs than pre-seizure epochs.

Patient (IEEG Portal)	Epoch Duration (Sec)	PS_0 (sec)	PS_1 (sec)	PS_2 (sec)	S_0 (sec)	S_1 (sec)	S_2 (sec)
HUP64_phaseII	107.0 ± 0.0	33 ± 0.0	31 ± 0.0	28 ± 0.0	42 ± 0.0	37 ± 0.0	27 ± 0.0
HUP65_phaseII	87.7 ± 4.5	24.0 ± 2.1	17.3 ± 3.8	15.0 ± 2.5	36.3 ± 2.3	27.0 ± 0.6	19.3 ± 2.7
HUP68_phaseII	96.8 ± 5.3	32.4 ± 6.5	24.8 ± 5.2	13.6 ± 2.3	32.3 ± 4.5	25.8 ± 3.5	21.0 ± 3.7
HUP70_phaseII	107 ± 0	4.9 ± 0.4	3.8 ± 0.5	1.8 ± 0.2	6.9 ± 0.7	3.0 ± 0.4	1.7 ± 0.3
HUP72_phaseII	90.0 ± 0.0	43.0 ± 0.0	23.0 ± 0.0	20.0 ± 0.0	12.0 ± 0.0	7.0 ± 0.0	6.0 ± 0
HUP73_phaseII	77.0 ± 4.7	23.0 ± 7.2	20.2 ± 6.3	10.4 ± 2.7	33.8 ± 2.3	31.2 ± 1.8	11.3 ± 3.1
HUP78_phaseII	46.4 ± 6.0	16.6 ± 4.6	10.8 ± 2.3	7.6 ± 1.0	18.2 ± 3.2	15.8 ± 2.9	4.6 ± 1.4
HUP79_phaseII	259 ± 0.0	96.0 ± 0.0	67 ± 0.0	59 ± 0.0	73 ± 0.0	68 ± 0.0	45 ± 0.0
HUP86_phaseII	51.0 ± 17.0	21.5 ± 3.5	20.5 ± 2.5	17.0 ± 1.0	19.5 ± 8.5	18.0 ± 8.0	15.5 ± 6.5
HUP87_phaseII	10.5 ± 1.0	11.5 ± 1.5	11.0 ± 2.0	5.5 ± 0.5	23.5 ± 7.5	21.0 ± 9.0	3.5 ± 2.5
Study 004-2	46.0 ± 0.0	9.0 ± 0.0	31 ± 0.0	28 ± 0.0	42 ± 0.0	37 ± 0.0	27 ± 0.0
Study 006	85.0 ± 14.0	30.0 ± 12.0	26.0 ± 13.0	12.5 ± 0.5	25.0 ± 1.0	18.5 ± 2.5	15.5 ± 1.5
Study 010	127 ± 8.0	38.0 ± 5.0	34.0 ± 3.0	14.0 ± 2.0	35.0 ± 9.0	33.0 ± 7.0	20.5 ± 3.5
Study 016	113 ± 18.8	22.7 ± 10.2	19.0 ± 8.5	12.0 ± 6.1	47.7 ± 7.2	32.7 ± 6.2	17.3 ± 8.1
Study 019	116.5 ± 16.3	41.3 ± 6.2	37.0 ± 6.5	13.7 ± 2.2	44.3 ± 6.3	38.3 ± 6.1	22.3 ± 4.2
Study 020	64.8 ± 25.9	6.8 ± 0.5	5.0 ± 0.4	3.5 ± 0.6	30.5 ± 12.4	24.5 ± 11.4	6.8 ± 3.8
Study 023	64.5 ± 11.0	17.3 ± 4.7	14.5 ± 3.7	11.3 ± 2.8	22.5 ± 6.9	18.3 ± 4.8	8.0 ± 0.0
Study 026	53.4 ± 2.3	14.2 ± 2.3	12.0 ± 2.0	7.0 ± 1.4	18.3 ± 1.3	15.0 ± 1.4	10.2 ± 1.3
Study 031	44.6 ± 3.9	19.2 ± 1.1	16.4 ± 1.2	6.5 ± 3.3	19.0 ± 2.9	16.2 ± 3.1	5.2 ± 1.0
Study 033	118 ± 31.4	45.1 ± 11.5	39.4 ± 9.1	28.0 ± 6.9	72.9 ± 14.0	60.1 ± 9.3	35.6 ± 8.5
Study 037	176 ± 56.0	31.5 ± 25.5	22.0 ± 18.0	19.0 ± 15.0	79.0 ± 20.0	56.5 ± 17.5	23.0 ± 14.0

Table A: **Event and state durations for patients.** Durations of epoch (pre-seizure / seizure) and states are averaged over all events in each patient; $\pm$ represent standard error.

References

1. Newman M, Girvan M. Finding and evaluating community structure in networks. Physical Review E. 2004 Feb;69(2):1–15. Available from: http://link.aps.org/doi/10.1103/PhysRevE.69.026113.
2. Newman ME. Modularity and community structure in networks. Proceedings of the National Academy of Sciences. 2006;103(23):8577–8582. Available from: http://www.pnas.org/content/103/23/8577.short.
3. Porter MA, Onnela JP, Mucha PJ. Communities in networks. Notices of the AMS. 2009;56(9):1082–1097. Available from: http://www.ams.org/notices/200909/rtx090901082p.pdf.
4. Fortunato S. Community detection in graphs. Physics Reports. 2010 Feb;486(3-5):75–174. Available from: http://linkinghub.elsevier.com/retrieve/pii/S0370157309002841.
5. Reichardt J, Bornholdt S. Statistical mechanics of community detection. Physical Review E. 2006 Jul;74(1). Available from: http://link.aps.org/doi/10.1103/PhysRevE.74.016110.
6. Fortunato S, Barthelemy M. Resolution limit in community detection. Proceedings of the National Academy of Sciences. 2007;104(1):36–41. Available from: http://www.pnas.org/content/104/1/36.short.
7. Mucha PJ, Richardson T, Macon K, Porter MA, Onnela JP. Community Structure in Time-Dependent, Multiscale, and Multiplex Networks. Science. 2010 May;328:876–878. Available from: http://www.sciencemag.org/cgi/doi/10.1126/science.1184819.
8. Bassett DS, Porter MA, Wymbs NF, Grafton ST, Carlson JM, Mucha PJ. Robust detection of dynamic community structure in networks. Chaos: An Interdisciplinary Journal of Nonlinear Science. 2013;23:013142. Available from: http://link.aip.org/link/CHAOEH/v23/i1/p013142/s1&Agg=doi.

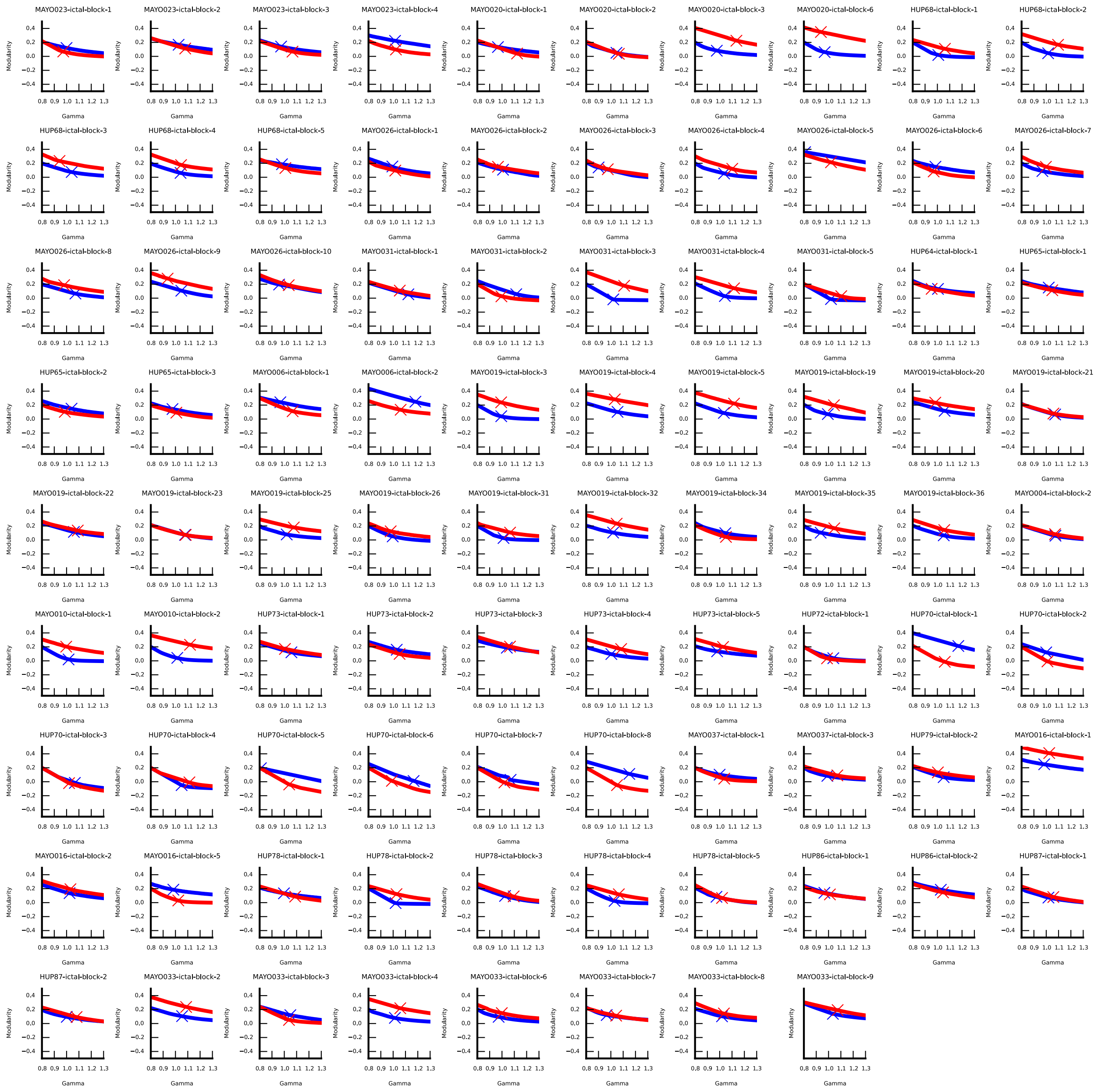

Figure A: **Structural Resolution Parameter Sweep.** Each graph demonstrates the effect of varying γ on modularity $\mathbf{Q}$ per epoch per patient. blue, pre-seizure; red, seizure. Crosses denote the γ chosen based on the inflection point for community detection results presented in the main manuscript.

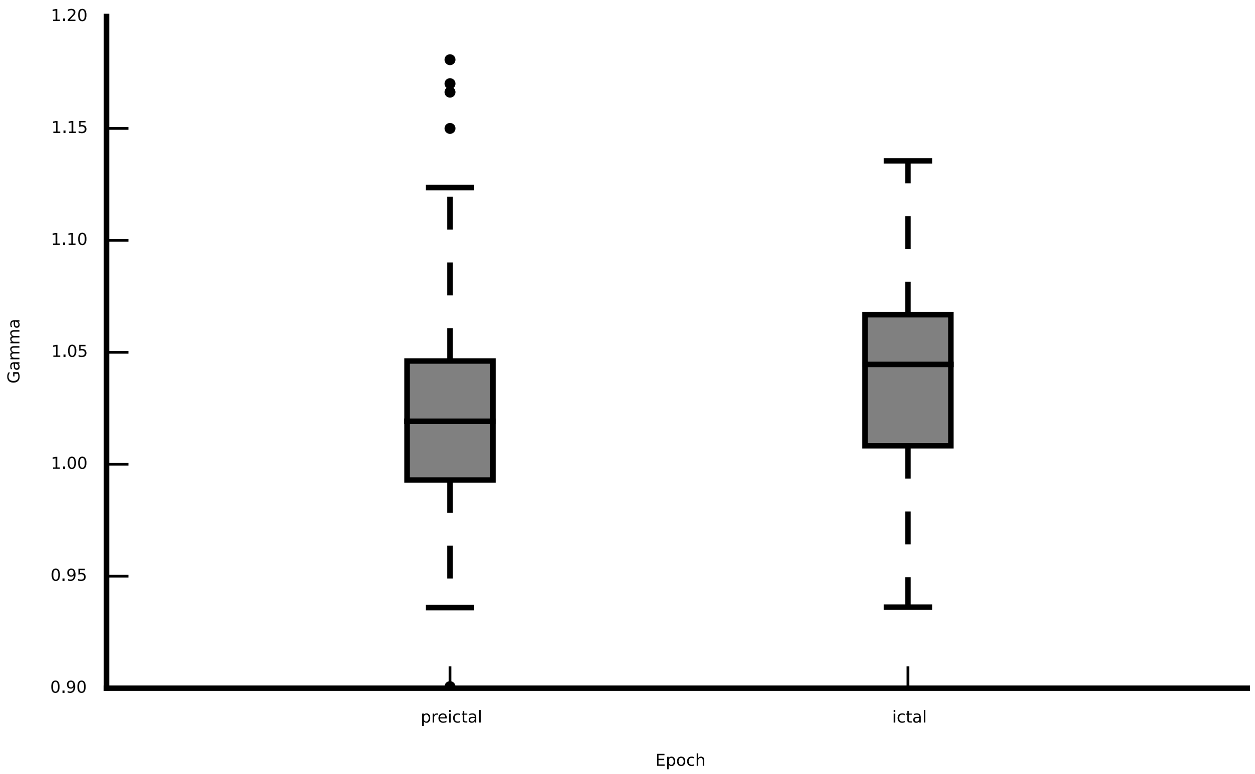

Figure B: **Distribution of Optimal Structural Resolution Parameter.** Optimal γ parameter for pre-seizure and seizure epochs ($N = 88$). On average, seizure epochs tend to have more granular temporal division.
